# Study on Optimal Parameter and Target for Pulsed-Field Ablation of Atrial Fibrillation

**DOI:** 10.3389/fcvm.2021.690092

**Published:** 2021-09-21

**Authors:** Xuying Ye, Shangzhong Liu, Huijuan Yin, Qiang He, Zhixiao Xue, Chengzhi Lu, Siying Su

**Affiliations:** ^1^The First Central Clinical College of Tianjin Medical University, Tianjin, China; ^2^Department of cardiology, Tianjin First Central Hospital, Tianjin, China; ^3^School of Biomedical Engineering and Technology, Tianjin Medical University, Tianjin, China; ^4^Institute of Biomedical Engineering, Chinese Academy of Medical Sciences & Peking Union Medical College, Tianjin, China; ^5^Tianjin Intelligent Health Medical Co., Ltd, Tianjin, China

**Keywords:** pulsed field ablation, irreversible electroporation, atrial fibrillation, pulmonary vein ablation, apoptosis

## Abstract

Pulsed-field ablation (PFA) had potential advantages in atrial fibrillation ablation, and we aim to confirm the optimal parameter and target of PFA for atrial fibrillation. Two ablation modes *in vitro* of single-cell system (ablation in electrode cup) and monolayer cell system (ablation in inserts with electrode tips) were established to perform PFA for myocardial cell H9C2 and smooth muscle cell A7r5. Ablation effect, calcium ion influx, the expression of Cx45, and surface morphological change were observed. Three Bama minipigs were used to verify the *in vivo* ablation effect of PFA. In monolayer cell system, H9C2 was significantly sensitive to PFA compared with A7r5, with shrinking of the whole monolayer. The ablation effect of bidirectional pulse was weaker than that of the two mono-polar pulses. Expressed Cx45 proteins were increased in H9C2 but decreased in A7r5 cells. Bidirectional PFA performed on Bama minipigs was able to effectively block electrical activity from the pulmonary vein to the atrium with week muscle contraction, not generating pulmonary vein stenosis. Bidirectional PFA was able to significantly ablate myocardial cells, maintain cell–cell connection, and reduce muscle contraction, which was a kind of optimized PFA strategy for atrial fibrillation.

## Introduction

Atrial fibrillation (AF) is a kind of common arrhythmia, with around 33.5 million patients globally. The incidence rate of AF is increased along the age. It is predicted that the number of AF patients will double until 2060. In 2019, the American Heart Association (AHA), American College of Cardiology (ACC), and Heart Rhythm Society (HRS) jointly updated the Treatment Guidelines for AF Patients (1) and pointed out that catheter ablation was the first-line therapy scheme for AF. The catheter ablations mainly include radiofrequency (RF) ablation and cryoballoon (CB) ablation. However, there is a zero-sum effect based on cold/hot ablation; overdose will cause complications including pulmonary vein stenosis, esophageal fistula, and phrenic nerve injury; underdose will cause incomplete isolation, and recurrence is likely to happen (2, 3), which limits the application of freezing/thermal catheter ablation.

Pulsed-field ablation (PFA) is a kind of ablation based on irreversible electroporation (IRE). It will form massive nanoscale membrane permeable holes on the cell membrane by virtue of the high-voltage direct current pulse emitted between electrodes (generally, the increasing and falling time of pulse is 200–800 ns; the pulse width is maintained for 5–100 μs) to lead to apoptosis of cells due to change of permeability (4). The non-thermal ablation of this way and selectivity of electric field intensity for different tissues will avoid damage to vessels and nerves during elimination of tumors (5, 6). The technique has been approved by the Food and Drug Administration (FDA) and National Medical Products Administration (NMPA) to be used for clinical treatment of liver cancer (6), pancreatic cancer (5), kidney cancer (7), and prostate cancer (8).

In recent years, the characteristic of selective ablation of PFA has attracted attentions from experts of cardiac electrophysiology to successively carry out multiple PFA cardiac ablation studies. Witt et al. (9) performed pulmonary vein ablation using catheter balloon IRE on five dogs and demonstrated irreversible transmural myocardial ablation without incidence of pulmonary vein stenosis. Koruth et al. (10, 11) compared the feasibility of RF and PFA in ablation in the pulmonary vein and superior vena vein and discovered that PFA could generate even specific ablation zone with clear boundary in myocardium, without evident damage to the nervous and venous structure. Reddy et al. (12) carried out phase I clinical trial for safety of PFA pulmonary vein ablation in two centers, and the result indicated that the success rate of 15 cases receiving catheter PFA pulmonary vein ablation was 100%, and the success rate of seven cases receiving epicardium PFA was 86%. Although it was proved by these researches that PFA had potential advantages in treatment of AF, the parameters of electric field of PFA varied greatly in different research reports; what is more, there was no comparative study on parameters of electric field, and PFA mechanism was unclear. The muscle convulsions associated with PFA are also a concern for the ablation of AF. Studies have shown that bidirectional pulses can reduce PFA-induced muscle twitches in the tumor ablation (13, 14), but no corresponding studies have been conducted in the application of AF ablation.

Therefore, in the study, we will focus on the comparison of PFA effect in different electric field modes and dose, to verify the optimal parameters for myocardial ablation, providing experiment evidence for PFA clinical treatment strategy planning of AF.

## Materials and Methods

### Materials

The high-frequency alternating PFA equipment, electrode needles, and ablation electrode catheter were developed by Tianjin Intelligent Health Medical Co., Ltd. (< city>Tianjin < /city>, China). Rat myocardial cell H9C2(2-1) and rat smooth muscle cell of thoracic aorta A7r5 were purchased from Cell Resource Center of the Institute of Basic Medical Sciences of the Chinese Academy of Medical Sciences (Beijing, China). Dulbecco's modified Eagle medium (8120286) culture mediums were purchased from GIBCO (Grand Island, NY, USA). Electrode cap Disposable Cuvettes (4-mm gap, 45-0126) were purchased from BTX (Holliston, MA, USA). Millicell® 24-well inserts (1-μm pore size, MCRP24H48) were purchased from Merck Millipore (Billerica, MA, USA). LIVE/DEAD® Viability/Cytotoxicity Kit (L3224) and Oregon Green® 488 BAPTA-1 AM (O6807) were purchased from Thermo Fisher Scientific (Waltham, MA, USA). Anti-TNF alpha antibody [EPR21753-109] (ab205587), Anti-Connexin 45/GJA7/Cx45 antibody [5B9.2] (ab78408), Goat Anti-Rabbit IgG H&L (Alexa Fluor® 488) ab150077, and Goat Anti-Mouse IgG H&L (Alexa Fluor® 647, ab150115) were purchased from Abcam (Cambridge, MA, USA). Hematoxylin and eosin/HE Staining Kit and Masson's Trichrome Stain Kit were purchased from Beijing Solarbio Science & Technology Co., Ltd. (Beijing, China). *In Situ* Cell Death Detection Kit, POD (11684817910) was purchased from Sigma-Aldrich (St. Louis, MO, USA).

### Cell Experiments

#### Cell Culture

The H9C2(2-1) and A7r5 cells were used for simulating the targets for PFA AF. DMEM-H complete medium (10% fetal calf serum, 4 mM of l-glutamine, 1% triple antibodies containing penicillin, streptomycin, and amphotericin B) was adopted for H9C2(2-1) cells; DMEM complete medium (10% fetal calf serum and 1% double antibodies containing penicillin and streptomycin) was adopted for A7r5 cells for routine culture. The culture environment was 5% CO_2_ 37°C incubator, with once passage every 2–4 days.

#### Pulsed-Field Ablation

Two PFA intervention modes were designed *in vitro*, including single-cell mode and monolayer mode.

### Single-Cell Mode

H9C2(2-1) cells or A7r5 cells in logarithmic phase were digested by trypsin and were made into cell suspension of 8 ^*^ 10^4^/ml concentration through centrifugation and resuspension; 600 μl of the suspension was added into the electrode cap as shown in (Supplementary Figure 1A) for PFA intervention according to the design parameters. Positive and negative anodes were at the two sides of the electrode cap, and the cell suspension was evenly distributed in the space electric field. After intervention, the cell suspension was re-inoculated to a 96-well-plate for 24 h for detection of cellular damage.

#### Monolayer Cell Mode

H9C2(2-1) cells or A7r5 cells in logarithmic phase were made into the cell suspension of the same concentration and inoculated into 24-well inserts. The cell culture mediums were added into both the inserts and the compartments under the inserts. Forty-eight hours later, PFA intervention was performed when monolayer cell membrane was formed. The intervention equipment is shown in Supplementary Figure 1B, equipped with four electrodes, including one positive electrode vertically suspended in the 24-well-inserts with electrode tip 2 mm away from cells, and three negative electrodes inserted to the basolateral compartment through three holes surrounding the inserts, with electrode tip 2 mm away from the well-bottom and 1 mm away from the insert basolateral. Conic electric discharge was formed between positive and negative electrodes. The electric field distribution on monolayer cell membrane was annular through simulation by COMSOL Multiphysics 5.5, and the strength of the electric field gradually increases outward from the center. Ablation was performed according to average field strength. After ablation, the monolayer cells were continued to culture for 24 h for survival detection of cells.

#### Grouping of Pulsed-Field Ablation Intervention

Three pulse modes were designed in the experiment, as shown in Supplementary Figure 1C, including bi-polar short pulse (forward pulse width was 5 μs with pulse interval of 3 μs; reverse pulse width was 3 μs with pulse interval of 5 μs), mono-polar short pulse (pulse width was 5 μs with pulse interval of 5 μs), and mono-polar long pulse (pulse width was 70 μs with pulse interval of 10 μs). Every 10 pulses were included in one group, with between-group interval of 1 s. In short pulse modes, PFA ablated 500 pulses, while in long pulse mode, PFA ablated 70 pulses. The effective acting time was 4,800–5,000 μs, and the field strength was set as 250, 500, 750, 1,000, and 1,250 V/cm.

#### Detection of Cell Ablation Effect

MTT [3-(4,5-dimethylthiazol-2-yl)-2,5-diphenyltetrazolium bromide] colorimetric method was adopted to detect ablation effect for the cells after PFA in single-cell mode. The details were as follows: in 24 h after ablation, 10 μl of MTT solution (5 mg/ml) was added into every well for continuous incubation for 4 h; the supernatant was carefully absorbed for discard; 100 μl of 50% sodium dodecyl sulfate (SDS)−50% DMF was added into the wells for dissolution for overnight; the optical density value (OD value) under 570 nm wavelength was measured through enzyme-linked immunometric meter. Cell survival rate was calculated by the following formula, in which S means survival rate.


$$\begin{array}{l} S=\frac{{OD}_{experimental\;group}}{OD_{control\;cell\;group}} \end{array}$$

Live/dead cell staining method was adopted to detect ablation effect for the cells ablated by PFA in monolayer cell mode. The details were as follows: in 24 h after ablation, cell staining was performed according to the protocol in the specification; after culture medium was discarded, 100 μl of mixed liquid containing 2 μM of calcein AM (for live cells) and 4 μM of EthD-1 (for dead cells) was added for staining for 30 min under room temperature; phosphate-buffered saline (PBS) was used to wash off the extra coloring agent; observation and photography were performed under confocal microscope. The excitation laser was 488 nm; 500- to 550-nm bandpass was adopted for calcein AM, and 600-nm longpass was adopted for EthD-1. IMAGEJ software was used to analyze the areas of both staining methods. EthD-1 could only stain the cell nucleus; thus, compensation was performed by multiplying the red staining area by 2. Cell survival rate was calculated according to the following formula, in which S means survival rate and A means area.


$$\begin{array}{l} S=\frac{A_{green}}{A_{green}+2A_{red}} \end{array}$$

#### Ca^2+^ Staining

Ten micromolar of OGB-1 (prepared with serum-free DMEM) was used to incubate the H9C2(2-1) cells and A7r5 cells growing into monolayer at both sides of inserts at 37°C for 1 h. After incubation, the serum-free medium was washed off, and new medium was added. Image capture in time-series mode was performed under confocal microscope immediately after PFA of cells in 500 V/cm field strength; λ_Ex_ = 488 nm and λ_Em_ = 490–545 nm; time interval was 10 s; 30 images were captured in total.

#### Scanning Electron Microscopy

Glutaraldehyde of 2.5% was used to fix monolayer H9C2(2-1) cells and A7r5 cells in insert 30 min and 24 h after PFA (500 V/cm), respectively. Gradient dehydration, drying, and metal spraying were performed. The cell surface appearance was observed under scanning electron microscopy (SEM).

#### Immunofluorescence Staining

Methyl alcohol was used to fix monolayer H9C2(2-1) cells and A7r5 cells in insert 2 and 24 h after PFA [500 V/cm for H9C2(2-1) and 750 V/cm for A7r5], respectively, for 10 min; and the Tris-buffered saline (TBS) containing 5% goat serum was used for blocking. Anti-Connexin 45 antibody (1:200) was incubated overnight at 4°C; after washing, fluorescent Goat Anti-Mouse IgG H&L (Alexa Fluor® 647) was incubated away from light at 37°C for 1 h; after washing again, the membrane of insert was carefully dissected, and the cells were placed on the glass slide upside down covered with coverslips for observation under confocal microscope. The condition for signal acquisition of Alexa Fluor® 647 was λ_Ex_ = 647 nm and λ_Em_ = 660 nm longpass.

### Animal Experiments

#### Ablation Procedure

Healthy Bama male minipigs (n = 3, 80 ± 10 kg) purchased from Tianjin Yuda Laboratory Animal Breeding Co., Ltd. (Tianjin, China) were fed conventionally. All experimental protocols involving pigs were approved by the animal ethics and welfare committee (approval number: 2017015) of Beijing Tonghe Shengtai Comparative Medical Research Institute, Beijing, China. Before operation, Lumianning (the compound preparation of Jingsongling, edetic acid (EDTA), DHE, and haloperidol) and midazolam injection were mixed by 1:1 and injected to the muscle according to 5 ml/kg (weight). 2,6-Diisopropyl-phenol injection was provided to maintain anesthesia at the speed of 5 ml/h. Skin preparation and sterilization were performed at the groin; femoral vein catheter was inserted under guidance by ultrasound; 8F catheter was inserted into the right atrium through the inferior caval vein with assistance of X-ray contrast radiography, penetrating the interatrial septum to reach the left atrium to find the pulmonary vein. The electrode was sent to the pulmonary vein along the catheter for PFA under 1,600 V/cm in bi-polar short pulse mode (the forward pulse width was 5 μs; reverse pulse width was 3 μs; pulse interval was 3 μs) with 1,000 pulses and 8-A current. ECG monitoring was performed during the operation. After ablation, pacing detection was performed in pulmonary vein and atrium to evaluate ablation effect. X-ray contrast radiography was performed for pulmonary vein to observe if there was spasm or bleeding. Hemostasis by compression was performed on the wound after the operation.

To verify the *in vivo* safety of PFA, PFA was performed on the renal artery of the experimental pigs under different field strengths after the pulmonary vein ablation. The 8F catheter entered the abdominal aorta through the femoral artery channel and then entered both sides of the renal arteries in turn with the assistance of X-ray contrast radiography. The ablation was performed at selected locations before the renal artery branches. The ablation dose of four renal arteries (two experimental pigs) was 1,000, 1,200, 1,600, and 2,000 V/cm, and two for control.

#### Detection of Blood Indexes

Blood sample was collected before the operation, 30 min and 72 h after the operation for detection of indexes including creatine kinase (CK), creatine kinase isoenzyme (CKMB), troponin (TNI), myoglobin (MYO), creatinine (CRE), low-density lipoprotein cholesterin (LDL-C), high-sensitivity C-reactive protein (hs-CRP), and N-terminal pro-brain natriuretic peptide (NT-pro-BNP).

#### Pathological Examination

Seventy-two hours after the operation, euthanasia (electric shock) was performed for the experimental pigs. Their heart tissues were taken for fixation and embedding to be as tissue slices. H&E staining, Masson's trichrome staining, and apoptosis staining were performed to observe ablation effect.

### Statistics

Origin8.5 software was used for data analysis. All the data were presented by χ±*SD*. Two-tailed *t*-test was adopted for between-group difference. One-way ANOVA was adopted to analyze multigroup difference. *p* < 0.05 was considered statistically significant.

## Results

### Pulsed-Field Ablation Effects on Cells

**Figure 1:**
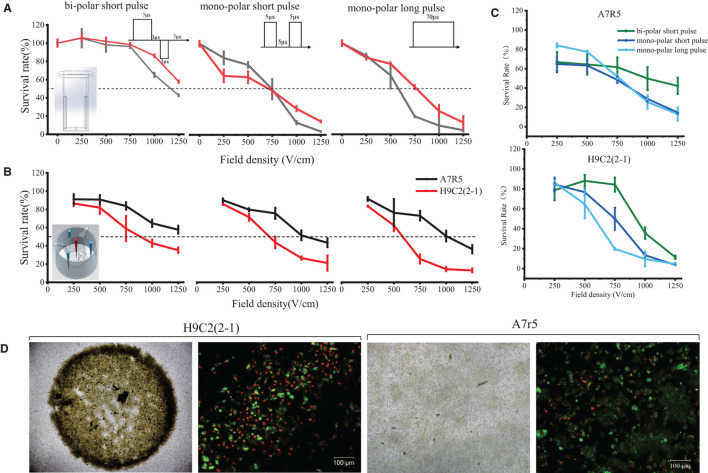
Dose–effect curves of PFA for H9C2(2-1) and A7r5 in single-cell and monolayer systems. **(A)** PFA on single cells. **(B)** PFA on monolayer cells. **(C)** Comparison of cell killing efficiency of three pulsed modes of PFA. **(D)** The morphologic change of cell monolayer after PFA. The top row shows the images under ×4 magnification; the bottom row shows the results of live/dead cell staining under ×10 magnification. PFA, pulsed-field ablation.

In order to screen the optimal PFA dose for clinic, two ablation systems and three ablation modes were designed in this study as shown in Supplementary Figure 1. In single-cell system, the field strength was evenly distributed, and PFA energy was received by single cells; while in monolayer system, the field strength was distributed annularly and was becoming weaker when approaching the center, and PFA energy was received by the whole cell monolayer simultaneously. The result is shown in Figure 1. In single-cell system (embedding graph of Figure 1A), the survival rate of H9C2(2-1) and A7r5 cells depended on the field intensity and pulse mode; cell viability was negatively correlated to the field intensity; i.e., the higher the field strength, the lower the cell survival rate. In the comparison of three pulse modes, the ablation effect of mono-polar short and long pulse was better than that of bi-polar short pulse; the lethal dose of 50% was around 750 and 1,250 V/cm (dotted line in the figure). There was no significant difference in sensitivity of H9C2(2-1) and A7r5 cells for PFA under the single-cell system. The difference was that, in monolayer cell system, although cell vitality still depended on the field intensity and pulse mode, there was significant difference in sensitivity of both cells for PFA; H9C2(2-1) was more sensitive to PFA than A7r5, and the difference was more significant when the field strength was larger. Compared with the three pulse modes (Figure 1C), the survival cells under the bidirectional pulse (green line) were slightly more than those of the unidirectional pulse (dark blue and light blue lines), but when the voltage was high enough, such as 1,250 V/cm, the ablation effect of the three pulse modes on H9C2(2-1) cells was similar. There was more significant difference in morphologic change of both cells after PFA. As shown in Figure 1D, H9C2(2-1) cell monolayer completely shrunk to the center after ablation, and the live/dead cells in the shrunk cell monolayer took their own proportion. However, disappearing cell–cell junction was found in A7r5 cells after PFA; single-cell shrunk *in situ* and live/dead cell staining indicated that massive cells were survived.

### Ca^2+^ Influx

**Figure 2:**
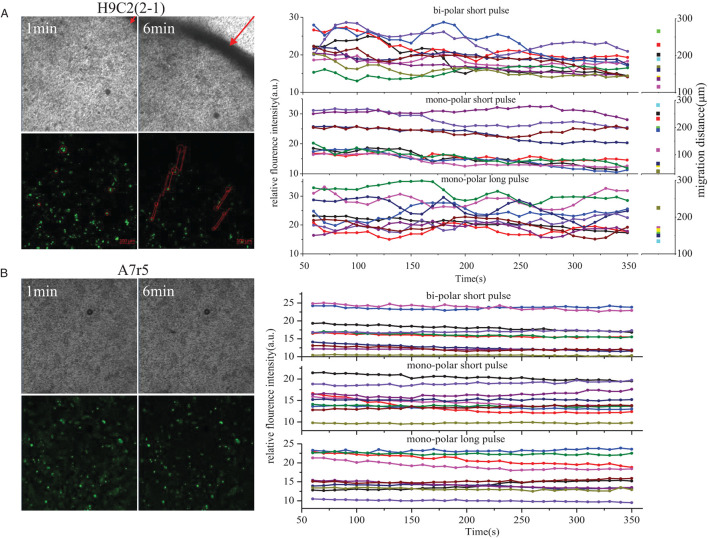
Ca^2+^ influx in H9C2(2-1)and A7r5 after PFA. **(A)** H9C2(2-1). **(B)** A7r5. PFA, pulsed-field ablation.

In order to better observe the different morphologic changes of H9C2(2-1) and A7r5 cells after PFA, Ca^2+^ fluorescent probe OGB-1 was used for cell incubation to observe the instant morphologic change of cells and Ca^2+^ influx after PFA. As shown in Figure 2A and Supplementary Video 1, the whole H9C2(2-1) cell monolayer shrunk to the center immediately after PFA, and the max displacement distance within the scanning period (300 s) was 266 μm. During rapid shrinking and displacement of cells, violent Ca^2+^ flash was found in cells, especially in bi-polar short pulse and mono-polar long pulse mode. Within 300 s, multiple peak values of Ca^2+^ fluorescence intensity were found, indicating multiple Ca^2+^ release and influx; it was also be affected by the movement of cells in and out of the focus during displacement. In mono-polar short pulse mode, shrinking happened to H9C2(2-1) cells as well, but with gentle fluctuation of Ca^2+^ fluorescence intensity. There was no significant difference in displacement distance of cells in the three modes (the scatter plot at the right of Figure 2A). Cell monolayer shrunk from peripheral region to the center, and the displacement distance of cells was greatly affected by the subjectivity in selection of visual field; thus, precise comparison failed. No evident displacement of A7r5 cells was observed, and amplitude of Ca^2+^ scintillation was significantly weaker than that of H9C2(2-1).

### Ultrastructure of Cell Membrane Perforation

**Figure 3:**
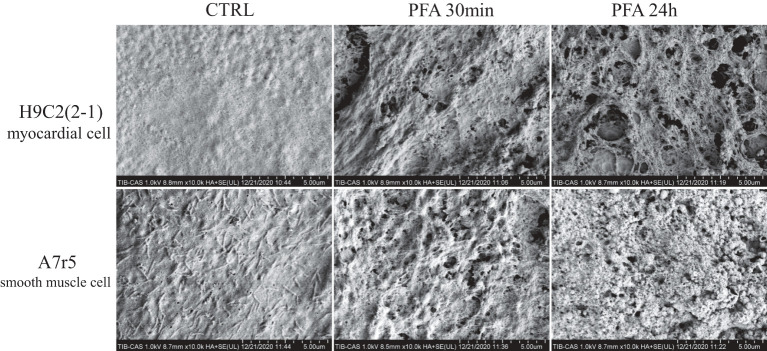
SEM images of H9C2(2-1) and A7r5 cells inoculated in transwell inserts 30 min and 24 h after PFA by ×10,000 magnification. SEM, scanning electron microscopy; PFA, pulsed-field ablation.

SEM was used to observe the electroporation on surface of myocardial cells and smooth muscle cells after PFA. As shown in Figure 3, the surface of H9C2(2-1) cells was smooth, with little pseudopod or villus. Thirty minutes after PFA, micropores of varied sizes were found on cell surface, around 30 micropores under ×10,000 magnification; 24 h later, the micropores on cell surface were enlarged, with fusion of multiple micropores. However, abundant pseudopods were found on surface of A7r5 cells; the pseudopods shrunk to a ball after PFA; massive electroporations were found on cell surface; 24 h later, electroporations were still there, and pseudopods shrunk to form a more evident shape of ball. Compared with the massive pseudopods on surface of A7r5 cells, surface of H9C2(2-1) cells was relatively smooth, which made it fail to closely cling to the transwell membrane. Such kind of ultrastructure explained the phenomenon of shrinking of H9C2(2-1) monolayer after PFA.

### Cx45 Expression

**Figure 4:**
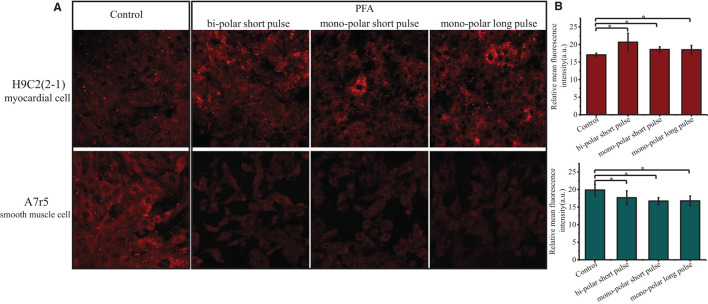
Effect of PFA on Cx45 expression in H9C2(2-1) and A7r5 cells. **(A)** Cx45 expression in H9C2(1, 2) and A7r5 cells 24 h after PFA under three modes. **(B)** Histogram of mean fluorescence intensity of Cx45 expression in H9C2(2-1) (top) and A7r5 (bottom) cells. *p < 0.05. PFA, pulsed-field ablation.

Cell–cell junction was the major difference between the two PFA systems *in vitro* (single-cell and monolayer modes) designed in this study. After observation of the fact that in cell monolayer system H9C2(2-1) was more sensitive to PFA than A7r5, and the shrinking of H9C2(2-1) monolayer cells, Cx45 (cell–cell connexin) expression in cells 24 h after PFA was detected. As shown in Figure 4, the baseline level of Cx45 expression in H9C2(2-1) cells was lower compared with A7r5. After PFA, with shrinking of cell monolayer, cell–cell junction was closer, and Cx45 fluorescence intensity was significantly reinforced; such phenomena were significantly evident under three modes compared with the control group, and enhancement under bi-polar short pulse mode was even significant, while Cx45 expression level in A7r5 cells was significantly reduced after PFA (*p* < 0.05), with significantly enlarged intercellular space.

### Pulsed-Field Ablation With Bi-Polar Short Pulse on Swine

**Figure 5:**
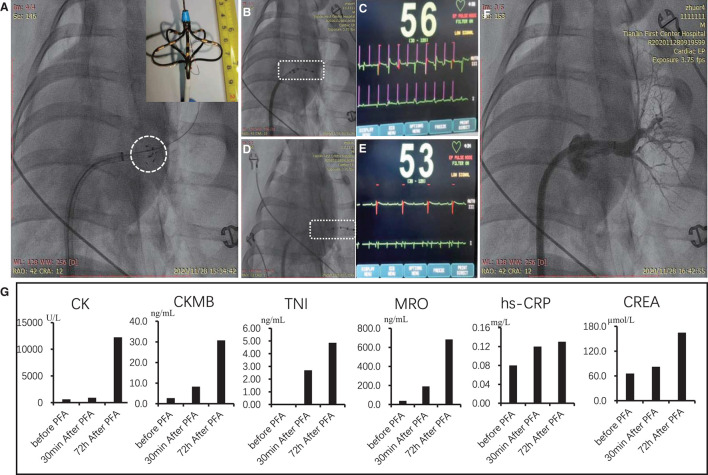
PFA of the left superior pulmonary vein of Bama minipigs. **(A)** Position and shape of PFA electrodes; the embedding graph is the enlarged view of electrode tip. **(B–E)** Effect of PFA verified by cardiac pacing. **(B)** Pacing electrode was in the non-injured part of atrium. **(C)** ECG image after pacing; the first line is pacing signal; the second line is ECG signal. **(D)** Pacing electrode was in the pulmonary vein for PFA. **(E)** ECG image after pacing. **(F)** radiographic image of the ablated pulmonary vein after PFA. **(G)** Indexes of myocardial damage and inflammatory reaction before PFA, 30 min and 72 h after ablation. PFA, pulsed-field ablation.

As shown in the embedding image of Figure 5A, the self-designed electrode device was in the shape of petal composed of five electrodes. Electric filed was formed between adjacent electrodes for sequential discharge. After PFA (1,600 V/cm) with 1,000 pulses, the manner of cardiac pacing was adopted to verify the instant effect of PFA. As shown in Figures 5B,C, when pacing electrode was in the left atrium (non-ablated area), the heart beats simultaneously; however, when pacing electrode was in the left superior pulmonary vein, which was ablated by PFA (Figures 5D,E), the heart failed to beat simultaneously, which indicated that the pacing signal in the left superior pulmonary vein was not conducted into atrial tissues, and PFA succeeded. Meanwhile, radiography showed that the structure of left superior pulmonary vein after PFA was intact, without spasm and bleeding, etc. (Figure 5F). During intraoperative observation, except mild muscle contraction of pig's chest and abdomen (see Supplementary Video 2) in the process of PFA, there was no other adverse reaction, and the pig was conscious in 30 min after operation.

### Pathological Changes of Atrial Tissues After Pulsed-Field Ablation

**Figure 6:**
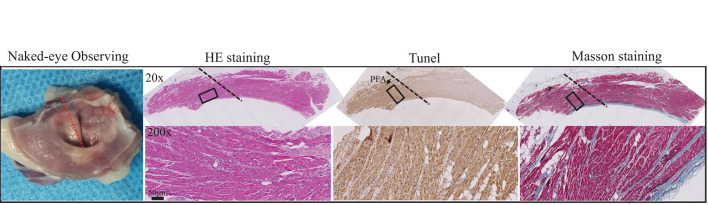
Pathological changes of myocardial tissues after PFA ablation by Gross observation, HE staining, Tunel staining and Masson staining from left to right. Black dotted lines represent the ablation boundary; the images in the black box are shown in the bottom row at 10 times magnification.

As shown in Figure 6 (left), annular ablated zone matching the ablation electrode was seen at the intersection of pulmonary vein and atrium, which was white necrosis-like completely different from the surrounding flesh-colored atrial tissues, with mild swelling. The ablated tissues were dissected along the direction of pulmonary vein for H&E staining, TUNEL staining, and Masson's staining to observe the pathological change. After H&E staining, evident boundary (presented by block dotted line) between ablated and non-ablated tissue was seen under ×20 magnification, and the space between cardiac muscle fibers in ablated tissue was enlarged; under ×200 magnification, the boundary was more clear, and in ablated tissue, the myocardial cell nucleus loss and massive inflammatory cells were infiltrating; ablation depth was transmural. In TUNEL staining, brown hyperchromatism was found in the ablated area, indicating that apoptosis of massive myocardial cells was caused by PFA. In Masson's staining, no significant change was found in the connective tissues between myocardial cells, and the structure of collagenous fibers was intact, infiltrated with massive inflammatory cells. These results indicated that PFA allowed controllable ablated area and transmural ablation depth (around 1 mm) and was able to cause massive apoptosis with mild inflammatory reaction.

In order to observe the safety of PFA, PFA was performed for the renal artery of the pigs under a range of field strengths. As shown in Supplementary Figure 2, PFA depth was gradually increased along increase of field strength. The ablation depth reached 4/5 renal arterial wall at 1,600 V/cm. When field strength reached 2,000 V/cm, ablation depth reached 1.45 mm, which was completely transmural, without any damage to the sympathetic nerve 1 mm away from the renal artery.

## Discussion

It has been demonstrated in multiple preclinical and clinical researches that PFA is applicable to ablation for AF. Its safety is superior to that of RF ablation due to its characteristic of thermal injury, which made it able to ablate myocardial tissues without damage to the surrounding esophagus and nerves, etc. (10–12). As a new ablation method, PFA is becoming the focus of AF ablation research (15). However, two problems emerge in the previous studies on PFA for AF: (1) Can PFA ablate myocardial tissue without damaging pulmonary veins due to field ablation? (2) Which ablation parameter is better for PFA, especially pulse parameters? There are different pulse parameters designed in reported studies, including pulse frequency from ns to ms, and pulse mode from bi-polar to mono-polar. To solve these problems, two comparative researches were performed in this study: comparison of response of H9C2(2-1) (simulating cardiac tissue) and A7r5 (simulating pulmonary vein) cells to PFA, and comparison of three pulse modes.

The onset and maintenance of AF are closely related to the rapid electrical activity of one or more lesions in the heart, the majority of which are distributed in the pulmonary veins. Thus, AF ablation usually blocks the site of origin of AF by establishing a barrier between the pulmonary vein and the atrium (16). However, ablation of pulmonary vein could cause the risk of stenosis. The best AF ablation method should be selective for cardiomyocytes. In order to verify the sensitivity of PFA to cardiomyocytes, the response of cardiomyocytes and smooth muscle cells to PFA was compared. We found that H9C2(2-1) not only was more sensitive to PFA than A7r5 but also has a completely different cell morphology. Both cardiomyocytes and smooth muscle cells have an electric field gradient dependence on PFA. When the electric field intensity is >1,000 V/cm, the injury of cardiomyocytes is sharply enhanced, while the injury of smooth muscle cells is <50% (Figure 1B), which fully demonstrates the high selectivity of PFA for cardiomyocytes. After PFA, H9C2(2-1) monolayer shrunk completely with intact cell–cell junction and high cell death rate (Figures 1B,C,2). To explain this phenomenon, we observed the Cx45 expression and the surface ultrastructure of monolayers, and we found that the pseudopod on the surface of smooth muscle cells seemed to have a protective effect against PFA injury. Fortunately, we first observed the overall ablation effect of PFA on monolayer cell inoculation conductive carriers. In previous studies, single-cell suspension was mostly used as study object because electrode cap was commercialized and easy for setting of ablation system. However, single cells cannot well-simulate the physiological activities of *in vivo* tissues, especially solid organs. Gianulis et al. attempted to study the ablation effect of adherent cells growing on glass coverslips (17). In order to increase the electrical conductivity, a coating of indium tin oxide was added to the non-cellular surface of the glass slides and then was placed into an electrode cup for ablation. But in fact, since glass is a poor conductor of electricity, the added conductivity of the coating makes it difficult to transfer to the cell layer. However, the monolayer system with inserts and electrode array we designed in the study have excellent electrical conductivity. Both sides of the cells are conductive medium, and the no-load resistance is very small. At the same time, the substrate membrane of the insert also simulated the cell matrix. The ablation system is closer to reality, and the membrane shrinkage after PFA of cardiomyocytes we found was also more able to reflect the real situation.

Pulse is the major parameter for PFA, including pulse width, pulse frequency, and pulse mode. Pulse width has been studied for ablation effects in several studies. Dermol-Cerne et al. compared the ablation effect for myocardial cells under ns, μs, and ms pulse width (18). Gianulis et al. studied the ablation effect of PFA for different cells under ns and μs pulse width (17). The μs pulse is generally considered to be more effective. And in the pulse mode, it was found that muscle contraction was likely to be induced by mono-polar pulse, which was one of the main side effects of PFA. However, when bi-polar pulse was adopted, the side effect of muscle contraction was significantly reduced due to depolarization of bi-polar pulse (13, 14). This is particularly important in the ablation of AF. Since the patient is under general anesthesia during tumor ablation, large amounts of muscle relaxants can be used to inhibit muscle convulsions caused by the unidirectional pulse (70–100 μs) PFA. However, AF ablation is performed under local anesthesia in some cases, so muscle relaxants cannot be used to relieve PFA muscle convulsions. Bidirectional pulses that reduce muscle twitching are more suitable for AF ablation than unidirectional pulses. The results of our cell experiments showed that although the damage of bidirectional pulse PFA on myocardial cells was slightly weaker than that of unidirectional pulse, the bidirectional pulse could still achieve the ideal ablation effect by increasing the voltage. Moreover, the effects of bidirectional and unidirectional pulses on the morphology, Ca^2+^ scintillation, and the expression of intercellular junction factor Cx45 were similar. These results suggest that bidirectional pulse can be used for the ablation of cardiomyocytes.

To verify the optimized result of PFA parameters in the cell experiment, *in vivo* experiments of pigs were also conducted in this study. We confirmed that PFA for the atrium by bi-polar short pulse can effectively block the conduction of electrical signals from the pulmonary vein to the atrium and can reduce the side effect of muscle contraction (Figure 5). Other findings including PFA-induced apoptosis and completeness of connective tissues, and the relationship between transmural effect and field strength, etc., were consistent with literature reports (19–21).

In conclusion, we found that PFA has a good ablation effect on both cardiomyocytes, which are more sensitive to PFA than smooth muscle cells. PFA can damage myocardial cells without destroying intercellular connections, which is beneficial to the maintenance of tissue structure. At the same degree of myocardial cell damage, the voltage required by bidirectional pulse was higher than that required by unidirectional pulse, but there were no differences in cell morphology, cell–cell injection, and Ca^2+^ activity. The *in vivo* experiment verifies the efficacy and safety of bi-polar short pulse PFA for myocardial cells. These findings answered questions about whether PFA can avoid pulmonary vein injury when ablating myocardial tissue, and the selection of electric field patterns (bidirectional electric field) and pulse parameters (>1,000 V/cm) of PFA. However, due to the small sample size of experimental pigs in this study and no observation of the long-term effects of PFA, more in-depth studies need to be continued. PFA is a novel technique that is promising for the treatment of AF ablation due to its non-thermogenic and selective nature of cardiomyocytes. In the process of clinical transformation, there are still a lot of challenges to be faced. Our team is committed to the research and development of PFA instrument for AF and the clinical transformation of AF PFA and will carry out more in-depth exploration on the basis of this study in the future.

## Data Availability Statement

The raw data supporting the conclusions of this article will be made available by the authors, without undue reservation.

## Ethics Statement

The animal study was reviewed and approved by the animal Ethics and Welfare Committee (approval number: 2017015) of Beijing Tonghe Shengtai Comparative Medical Research Institute, Beijing, China.

## Author Contributions

ZX and CL: conceptualization. XY, SL, QH, and SS: methodology and investigation. XY: data curation. HY: writing—original draft preparation and visualization. ZX: writing—review and editing and funding acquisition. All authors have read and agreed to the published version of the manuscript.

## Funding

This research was funded by Tianjin Science and Technology Committee (Grant Nos. 19ZXYXSY00050 and SQ2020YFF0406649) and Tianjin Municipal Health Bureau (Grant No. ZC20165).

## Conflict of Interest

SS was employed by the company Tianjin Intelligent Health Medical Co., Ltd. The remaining authors declare that the research was conducted in the absence of any commercial or financial relationships that could be construed as a potential conflict of interest.

## Publisher's Note

All claims expressed in this article are solely those of the authors and do not necessarily represent those of their affiliated organizations, or those of the publisher, the editors and the reviewers. Any product that may be evaluated in this article, or claim that may be made by its manufacturer, is not guaranteed or endorsed by the publisher.
