# Supplementary material for: Study on Optimal Parameter and Target for Pulsed-Field Ablation of Atrial Fibrillation
Source: Front Cardiovasc Med. 2021 Sep 21;8:690092. doi: 10.3389/fcvm.2021.690092 (PMC8490619; doi:10.3389/fcvm.2021.690092)
Supplement: Supplementary file 1 [file Data_Sheet_1.DOCX]

Supplementary Material

# PFA ablation system and parameter setting

To truly simulate the in vivo ablation effect, two PFA systems were designed in this experiment, as shown in Figure S1.


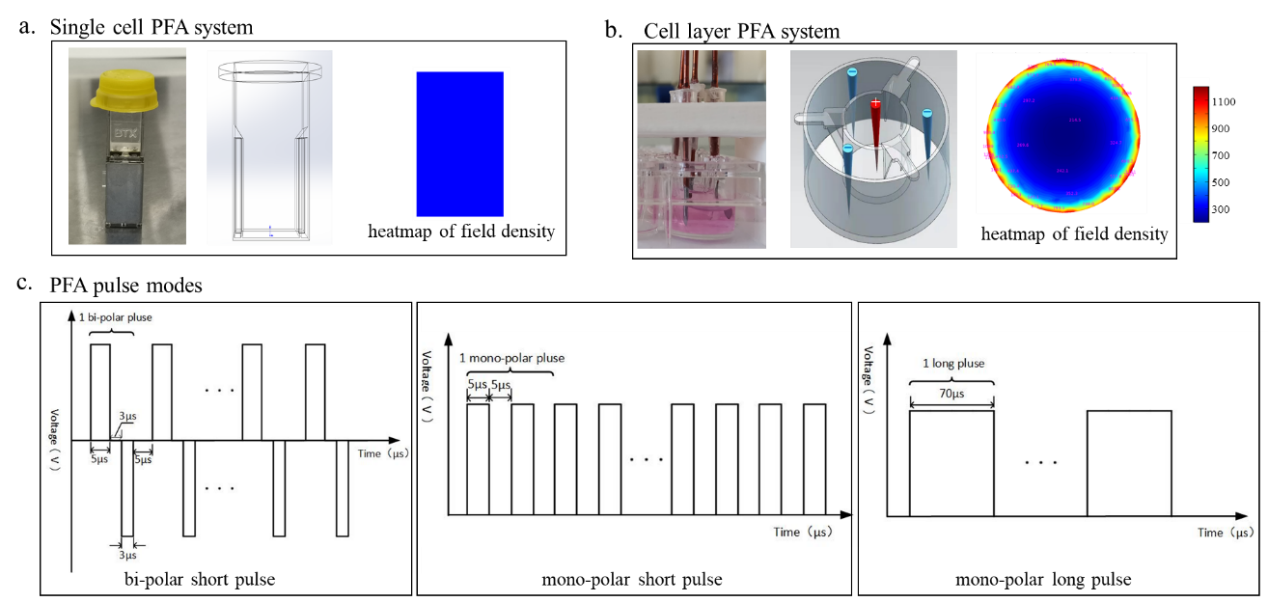


Supplementary Figure S1. PFA systems and pulse settings. (A) In the single-cell PFA system, a commercialized electrode cup (left) was used. The positive and negative electrodes at both sides of the electrode cup are shown in the schematic diagram (middle). Simulation results showed the electric field strength was even (right) between the two electrodes. (B) In the cell monolayer PFA system, cells were inoculated onto the membrane of the culture insert (left) to form a cell monolayer with cell–cell junctions. The self-made positive and negative electrodes were arrayed in and out of the inserts, respectively. The pulsed electrical field penetrated the membrane of the insert (cell monolayer) vertically (middle). The simulated diagram of electrical field strength (right) shows that the field strength was distributed annularly and became stronger from the center to the periphery. (C) PFA pulse mode: three types of pulse mode were used, including a bi-polar short pulse (left) with alternative positive and negative electric fields (the forward pulse width was 5 µs with a pulse interval of 3 µs, and the reverse pulse width was 3 µs with a pulse interval of 5 µs); a mono-polar short pulse (middle) (the pulse width was 5 µs with a pulse interval of 5 µs); and a mono-polar long pulse (right) (the pulse width was 70 µs with a pulse interval of 10 µs). Every 10 pulses were included in one group, with an interval of 1 s in every group.

# PFA induced shrinking of myocardial cellular monolayer

PFA induced shrinkage of the myocardial cell monolayer towards the center. Thirty slices captured in time-series mode after PFA in bi-polar short-pulse mode were made into a movie (Movie S1).

Supplementary Video S1. Shrinkage of the myocardial cell monolayer induced by PFA. With the cell monolayer PFA system, H9C2(2-1) cells were pre-incubated with the Ca2+ probe, OGB-1, and observed under a confocal microscope immediately after ablation. Scanning by time-series was performed with a time interval of 10 s, and 30 images were obtained and exported into a movie. After ablation, the cell monolayer rapidly shrunk from the peripheral region of the chamber to the center. Three representative cells were selected to present the displacement distance (red straight lines).

# PFA induced mild muscle contraction

In animal experiments, PFA was performed at the junction of the left atrium and the pulmonary vein in bi-polar short-pulse mode. The process of ablation is shown in Movie S2.

Supplementary Video S2. PFA of the heart in Bama miniature pigs. Under the assistance of X-ray radiography, the electrode was sent into the junction of the left atrium and pulmonary vein through the femoral vein for ablation at 1600 V/cm. During ablation, mild ablation of muscle was found, and the experimental pig demonstrated stable respiration and a steady heart rate.

# Depth of PFA ablation

To verify the *in vivo* safety of PFA, PFA was performed on the renal artery of experimental pigs under different field strengths (Figure S2). With an increase in field strength, PFA depth gradually increased. The ablated area of the vascular wall was significantly thinner compared with the non-ablated area, but muscle fibers were intact at 1000 V/cm. When 1200 V/cm was reached, partial muscle fibers in the ablated area of the vascular wall were replaced by collagenous fibers, and the ablation depth was around 2/3 of the thickness of the vascular wall. The ablation depth reached 4/5 of the renal arterial wall when it increased to 1600 V/cm. Completely transmural renal arterial wall was realized at a field strength of 2000 V/cm, and the ablation depth reached 1.45 mm, without any damage to sympathetic nerves 1 mm away from the renal artery.


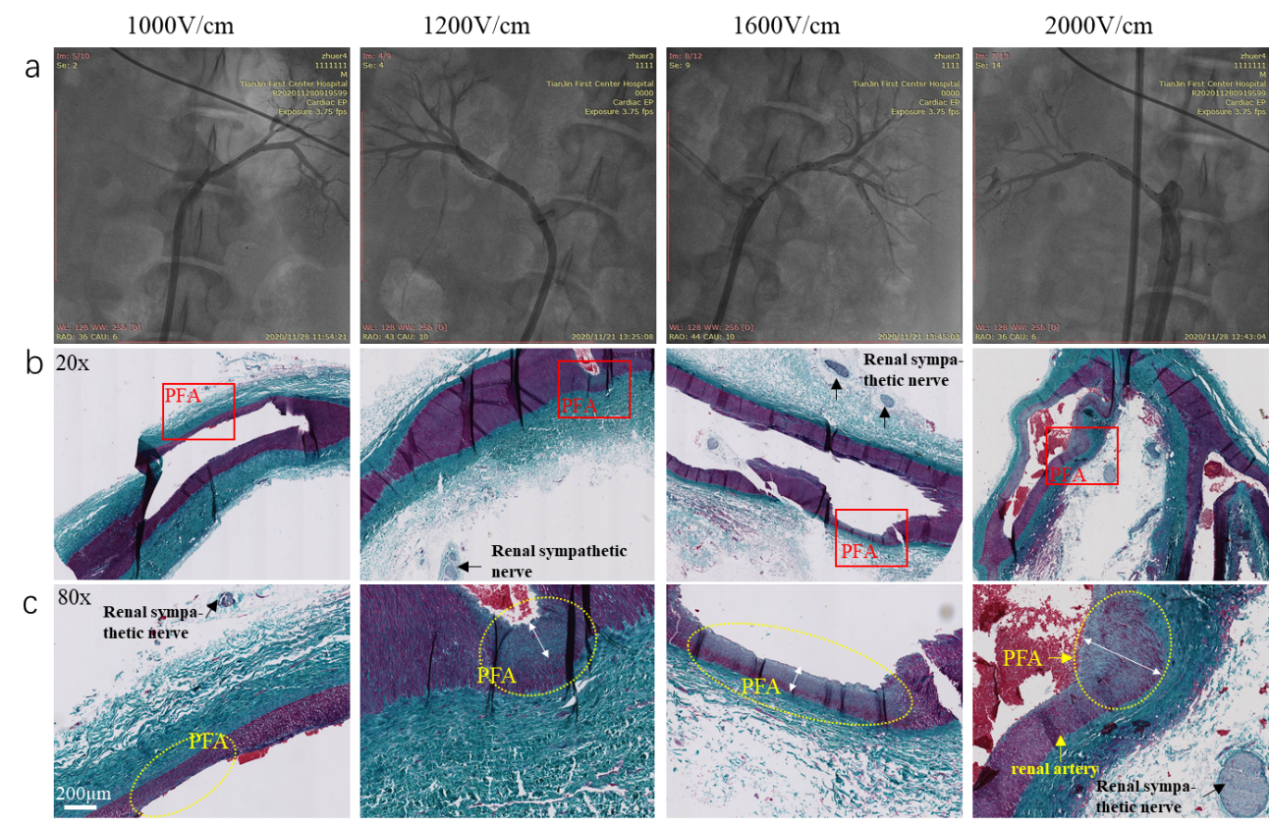


**Supplementary Figure S2.** Dose–effect of PFA of the renal artery. (A) PFA was performed on the left and right renal arteries of different experimental pigs using a series of field strengths (1000–2000 V/cm, respectively) with the assistance of X-ray radiography, and bi-polar short-pulse mode (5-3-3-5) was used. (B) On the third day after ablation, experimental pigs were euthanized, and the renal arteries were used to prepare tissue slices for Masson’s trichrome staining (20× magnification). Red represents muscle fibers, and blue represents collagenous fibers. The red frame shows the ablated area (80× magnification). (C) The yellow circle shows the ablated area, and the white double-headed arrow indicates the ablation depth 。
